# Friendly touch increases gratitude by inducing communal feelings

**DOI:** 10.3389/fpsyg.2015.00815

**Published:** 2015-06-15

**Authors:** Cláudia Simão, Beate Seibt

**Affiliations:** ^1^Centro de Investigação e Intervenção Social, Instituto Universitário de LisboaLisbon, Portugal; ^2^Department of Psychology, University of OsloOslo, Norway

**Keywords:** gratitude, touch, communal relationships, liking, relational models theory

## Abstract

Communion among people is easily identifiable. Close friends or relatives frequently touch each other and this physical contact helps identifying the type of relationship they have. We tested whether a friendly touch and benefits elicit the emotion of gratitude given the close link between gratitude and communal relations. In Study 1, we induced a communal mindset and manipulated friendly touch (vs. non-touch) and benefit to female participants by a female confederate. We measured pre- and post-benefit gratitude, communal feelings, and liking toward the toucher, as well as general affect. In Study 2, we manipulated mindset, friendly touch and benefit, and measured the same variables in female pairs (confederate and participants). In both studies the results showed a main effect of touch on pre-benefit gratitude: participants who were touched by the confederate indicated more gratitude than those not touched. Moreover, benefit increased gratitude toward a confederate in the absence of touch, but not in the presence of touch. Additionally, perceiving the relationship as communal, and not merely liking the confederate, or a positive mood mediated the link between touch and gratitude. The results further support a causal model where touch increases communal feelings, which in turn increase gratitude at the end of the interaction, after having received a benefit from the interaction partner. These results support a broader definition of gratitude as an emotion embodied in communal relationship cues.

## Introduction

Gratitude can trigger the need for physical contact. When one feels grateful, it is well accepted to hug the benefactor as a way to express gratitude (Algoe and Haidt, 2009). However, the opposite also seems to be true: Being hugged can trigger feelings of gratitude. The man who started the worldwide known “Free Hugs Campaign” based this action on being hugged by a stranger, at the right moment. He described that moment as the greatest thing that ever happened to him. Not surprisingly, hugging is perceived as one of the most central and positive features of gratitude (Lambert et al., 2009). However, to date, there is no experimental evidence that physical contact leads to feelings of gratitude. The present study therefore tested this causal link and the mechanism behind it. We suggest that physical contact embodies a communal relation and therefore increases gratitude for the relation.

Communal relationships are characterized by strong ties among individuals, following an “all for one and one for all” principle (Fiske, 1992). Communal partners feel intimate to each other and physically close. Mental representations of communal relations are based on the conception that bodies are the same or connected in some essential respect (Fiske and Haslam, 2005). Therefore, bodily proximity and friendly touch are used to communicate communal feelings. This communication serves to align the relational models of interaction partners in order to improve social coordination (Fiske, 1992).

Accordingly, being touched in a friendly way, as an embodied cue for a communal sharing model, should lead to experiencing the relationship as communal (Fiske and Haslam, 2005; Schubert et al., 2008). Previous research has shown that touch increases reliance on one’s teammates at the expense of one’s individual performance in basketball (Kraus et al., 2010) as well as cooperative behavior in a public goods game (Kurzban, 2001), and that it is used to communicate prosocial intentions among interaction partners (Hertenstein et al., 2006). These findings support our hypothesis because team play, public goods games, and prosocial behavior are all examples of the “all for one and one for all” principle of communal sharing (e.g., Fiske, 1992). Therefore, we predict that the unobtrusive friendly touch of an interaction partner augments communal feelings toward her in a dyadic situation with minimal direct interaction.

Furthermore, gratitude and communal relationships are highly related to each other. This link can have different reasons: communal relations are experienced as something valuable, thus something to be grateful for (Lambert et al., 2009; Gordon et al., 2010). Communal partners are responsive to each other’s needs (Clark and Mills, 1979; Clark, 1984; Clark et al., 1998; Clark and Jordan, 2002; Mills et al., 2004) and the reassurance of being taken care of by the communal partner can induce gratitude (Clark, 1984; McCullough et al., 2001; Algoe et al., 2008, 2010; Algoe and Haidt, 2009; Lambert et al., 2010). The *find-remind-and-bind-theory* posits that “within the context of reciprocally-altruistic relations, gratitude signals communal relationship norms” (Algoe, 2012, p. 455). Thus, according to this theory, it is the identification of a high quality communal relationship rather than the presence of a concrete benefit that triggers gratitude. Additionally, even when concrete benefits trigger gratitude (e.g., McCullough et al., 2008), this may be limited to situations in which the relationship to the benefactor is seen as communal, as recent evidence suggests (Simão and Seibt, 2014): Across different situations, benefits increased gratitude to the extent to which participants applied a mental model of a communal relationship to an interaction. Furthermore, applying this mental model predicted later gratitude in the absence of any concrete benefit. Thus, if gratitude is dependent on perceiving the relationship as communal, then cues which communicate communal intentions (e.g., touch) should be sufficient to activate gratitude toward the toucher.

Accordingly, we predicted that a friendly touch triggers gratitude. Because touch sends communal signals (e.g., to comfort or to bond; for a review, see Gallace and Spence, 2010), and relationships perceived as communal increase feelings of gratitude (Simão and Seibt, 2014), we hypothesized that feeling communal toward an interaction partner would mediate the link between touch and gratitude.

Moreover, a friendly touch also increases the likelihood of evaluating the toucher as more positive (Hornik, 1992). A request accompanied by a friendly pat on the shoulder leads to greater compliance than without the pat (Smith et al., 1982; Guéguen, 2002). This is particularly true when the toucher is liked rather than disliked (Baron, 1971). A brief hand-to-hand touch by a library clerk increases liking and positive feelings for the clerk (Fisher et al., 1976). Accordingly, we predicted that a friendly touch would increases liking for the toucher and positive affect, but neither liking nor positive affect would be sufficient to augment gratitude. Liking for another person is not specific to communal relationships (Clark and Mills, 1979), and therefore should not trigger gratitude. Gratitude is felt toward a partner who is experienced as being responsive to one’s needs (Algoe et al., 2008), and communal relationships rely on this relational responsiveness (Reis et al., 2004), which in turn promotes well-being (Fredrickson, 1998). Thus, gratitude should have different preconditions than liking for another person or positive affect: gratitude depends on feeling communal toward someone, appreciating the presence of him/her in one’s own life (Lambert et al., 2009).

### Overview of the Studies

Across two studies, we test if communal cues increase gratitude, and if this effect is mediated by communal feelings. Even though no statistical model can prove causality, assumptions about causality, which are logically derived from a theoretical argument, can be tested with a research design assessing the variables in the appropriate temporal sequence (Hayes and Preacher, 2014). We therefore test a causal sequence in which a friendly touch increases communal feelings (e.g., Fiske, 1992; Schubert et al., 2008), which in turn predict later gratitude (Simão and Seibt, 2014). The corresponding mediation analyses can be interpreted causally if a model where mediator and outcome are reversed is not also significant (Hayes and Preacher, 2014). In both studies, a female confederate of the experimenter touched (vs. did not touch) the participant briefly on the shoulder. We predicted that a friendly touch would increase feelings of gratitude, and that a feeling of communion toward the confederate (and not liking or positive affect) would mediate this link.

Furthermore, given that benefits can induce gratitude (e.g., McCullough et al., 2001), we explored whether receiving a benefit moderates the effect of a friendly touch on gratitude. Thus, after completion of the dependent variables, the confederate benefited the participant, and we measured gratitude again. We predicted that receiving a benefit would increase gratitude in the absence of a friendly touch. Yet, if gratitude depends on communal cues, then a friendly touch should suffice to produce gratitude, independent of whether one also receives a concrete benefit.

## Study 1

### Materials and Methods

In Study 1, participants first answered a questionnaire about friendship (Schubert and Giessner, in preparation), in order to ascertain an interpretation of the following touch as friendly and communal. Then, they completed an alleged team task with a confederate, whereupon half were touched by her. After completing the dependent measures, all participants received the benefit, and indicated their gratitude again.

### Participants

Thirty-six Portuguese female participants with a mean age of 20.23 (SD = 2.00) contributed to the current study, and all fulfilled our pre-defined inclusion criteria (see Supplementary Table S1). Participants were all students from a Portuguese University. We decided on the sample size based on the reported effect size of similar studies (e.g., Hornik, 1992). Thus, in order to have 80% power and to detect the estimated effect size (*d*) of 0.99, we aimed for a sample size of 36 participants^1^. All procedures were conducted according to the ethical guidelines and approved by the ethics board of the Scientific Commission of the hosting institution, Centro de Investigação e Intervenção Social (CIS-IUL).

### Dependent Variables

All dependent variables were assessed on Likert-type scales from 1 (not at all) to 7 (extremely). Descriptive statistics can be found in **Table 1**.

**Table 1:** Means and SD as a function of touch, and correlations of pre- and post-benefit gratitude, positive and negative affect, communal feelings, and liking indices of Study 1 (*N* = 36).

Variables	Touch Mean (SD)	No touch Mean (SD)	1	2	3	4	5
1. Pre-benefit gratitude	3.67 (1.57)	2.28 (1.36)	–				
2. Post-benefit gratitude	3.44 (1.82)	3.06 (1.47)	0.58^∗∗∗^	–			
3. Positive affect	3.86 (1.30)	2.71 (1.34)	0.76^∗∗∗^	0.41^∗^	–		
4. Negative affect	1.34 (.61)	1.58 (1.01)	0.17	0.23	0.11	–	
5. Communal feelings	3.99 (1.31)	3.15 (0.90)	0.66^∗∗∗^	0.50^∗∗^	0.58^∗∗∗^	0.03	–
6. Liking index	4.67 (1.19)	3.78 (1.06)	0.56^∗∗∗^	0.43^∗∗^	0.39^∗^	-0.08	0.70^∗∗∗^

^∗^*p* < 0.05; ^∗∗^*p* < 0.01; ^∗∗∗^*p* < 0.001.

#### Gratitude, Positive, and Negative Affect

After the touch (vs. no-touch) and again after the benefit, participants received measures of gratitude, positive and negative affect in randomized order, to disguise the purpose of the experiment. For gratitude, the item was: “*To what extent did you feel gratitude regarding your team partner?*” (*pre-benefit gratitude* and *post-benefit gratitude*). For affect, participants answered five positive^2^ (*interested, enthusiastic, determined, inspired, and excited*, α = 0.92) and five negative (*distress, upset, irritable, scared, hostile*, α = 0.89) items from the Portuguese adaptation of the Positive and Negative Affect Scale (Galinha and Pais Ribeiro, 2005).

#### Communal Feelings Index

To assess communal feelings, we used two scales in combination. Both scales measure slightly different aspects of communal feelings, so by combining them, we obtained a more valid and reliable measure of it. The two scales were Haslam’s (1994) communal sharing scale (e.g., “*What’s mine is yours’ would be true in this relationship*”), and Lakens and Stel’s (2011) *entitativity* and *rapport* scales, concerning the perception of the team as a social unit (e.g., “*I experience a feeling of togetherness between the individuals in this team*”), and the extent to which individuals were feeling connected (e.g., “*To what extent did both of you feel the same*”; 16 items, α = 0.94).

#### Liking Index

We computed a *liking index* comprised of one item measuring the perception of the confederate as *nice* (*To what extent do you consider your team partner as nice*) and one item related to *warmth* (*To what extent do you consider your team partner as warm; r* = 0.82, *p* < 0.001).

### Procedure

Participants arrived at the lab to participate in a teamwork experiment. The confederate waited near the lab room, allegedly previously recruited for the pair. Both were invited to sit down in a small room, seated at separate work stations facing opposite directions. After reading and signing the consent form, they started the experiment on two different computers, where the study was introduced as being about teams. To induce a communal mindset, participants were instructed to write down five sentences or words to describe friendship, as follows (adapted from Schubert and Giessner, in preparation):

This short survey is about the perception of friendships. We want to know how individuals perceive friendships. In this questionnaire, we will ask you to give a short description about what you think friendship is about. Afterward, we will ask you to perform an online team task and to answer some questions about your team partner, whether this person could be a good friend of you. Research suggests that with only a little information, we can create concrete assumptions and evaluations about people. Therefore, we ask you to answer all questions, even if you think that you do not have enough information about your team partner. There are no right or wrong answers for any of the questions you will be presented with; we are only interested in your opinion. Can you tell us what you think is a good friendship? What defines, in your opinion, a friendship? You can type sentences or keywords. Try to think of the five most important things or facts that define friendship.

No limits regarding time or text length were given. When participants were done, they saw an instruction to call the experimenter. She instructed them when to start the next task: a line bisection task (distractor task), which was allegedly done concurrently with the other participant. Participants were told to read the instructions on the screen and to start the task as soon as they were ready. In the written instructions participants were told to “complete the subsequent task by marking what you perceive as the midpoint of each of 10 horizontal lines, presented on the computer screen. In order to succeed, both team members must correctly indicate the midpoint of each line.” Thirty seconds after starting the distractor task, the experimenter left two pieces of paper on the confederate’s table. In the touch condition, the confederate touched the shoulder of the participant, who was facing the other way, for 1–2 s, and gave the participant one of the two pieces of paper. In the no-touch condition, the confederate placed one piece of paper on the participant’s table. Upon completion of the distractor task, participants filled out the gratitude item, the communal and the liking scales. Afterward, an instruction appeared to call the experimenter.

The experimenter explained that the main team task was a quiz. This quiz would be used to understand teamwork, how people construct networks, and how they accomplish goals when working together. The piece of paper they had received contained several topics for a quiz, and individually they should mark which topic they liked the most and which they liked the least. The quiz topic would be selected according to their preference. Then, the experimenter took both pieces of paper, and announced that the preferred topic of one of them (confederate), was the least liked topic of the other (participant). The confederate intervened asking the participant which topic she preferred. After her response, the confederate agreed to take that topic (benefit given to participant). Both were instructed to turn to their computers and to finish the questionnaire, so they could start the quiz. They filled out the post-benefit gratitude item, the manipulation check for benefit, and some demographics. Then, participants were probed for suspicion (see Supplemental Material), fully debriefed, and paid with a €5 gift voucher.

### Results

#### Touch and Pre-Benefit Gratitude

To test our specific hypothesis that receiving a friendly touch increases gratitude without any concrete benefit, we conducted a univariate ANOVA. We tested whether merely receiving a friendly touch on the shoulder increased the self-reported gratitude toward the confederate. We entered the pre-benefit gratitude as the dependent variable and the condition (touch vs. no touch) as the between-subjects factor. As predicted, there was a significant main effect of touch on pre-benefit gratitude, *F*(1,34) = 8.02, *p* = 0.008, η^2^ = 0.19. In the touch condition, participants reported feeling more grateful toward the confederate [*M* = 3.67, SD = 1.57, 95% CI = (2.96; 4.37)] than in the no touch condition [*M* = 2.28, SD = 1.36, 95% CI = (1.57; 2.98)].

#### Touch, Communal Feelings, Liking, and Affect

We then tested the effect of touch on the communal feelings, liking, and affect indices. Results showed a significant effect of touch on the communal feelings index, *F*(1,34) = 5.16, *p* = 0.030, η^2^ = 0.13. Participants in the touch condition perceived the relationship with the confederate as more communal [*M* = 3.99, SD = 1.30, 95% CI = (3.46; 4.53)] than did participants in the no-touch condition [*M* = 3.15, SD = 0.90, 95% CI = (2.61; 3.68)]. Touch also significantly increased liking, *F*(1,34) = 5.61, *p* = 0.024, η^2^ = 0.14. Participants who received a touch on the shoulder liked the confederate more [*M* = 4.67, SD = 1.19, 95% CI = (4.13; 5.21)] than participants who did not receive a touch on the shoulder [*M* = 3.78, SD = 1.06, 95% CI = (3.24; 4.32)].

For affect, we found a main effect of touch on positive affect, *F*(1,34) = 6.78, *p* = 0.014, η^2^ = 0.17. Participants in the touch condition felt more positive affect [*M* = 3.86, SD = 1.30 95% CI = (3.22; 4.49)] than participants in the no-touch condition [*M* = 2.71, SD = 1.34, 95% CI = (2.08; 3.34)]. The effect of touch on negative affect was not statistically significant, *F* < 1, *p* = 0.41.

#### Touch, Communal Feelings, and Post-Benefit Gratitude

##### Benefits and gratitude

In order to explore whether receiving a benefit moderates the effect of touch on gratitude, we conducted a repeated measures GLM with benefit (pre- and post-benefit gratitude) as the within-subjects factor, and touch as the between-subjects factor. Touch increased overall gratitude: participants in the touch condition reported higher levels of overall gratitude [*M* = 3.56, SE = 0.33, 95% CI = (2.89; 4.23)], than participants in the no-touch condition [*M* = 2.67, SE = 0.33, 95% CI = (2.00; 3.34)], however this main effect was not significant, *F*(1,34) = 3.64, *p* = 0.065, η^2^ = 0.10. The main effect of benefit on overall gratitude was also not statistically significant, *F*(1,34) = 1.38, *p* = 0.248, η^2^ = 0.04.

**FIGURE 1:**
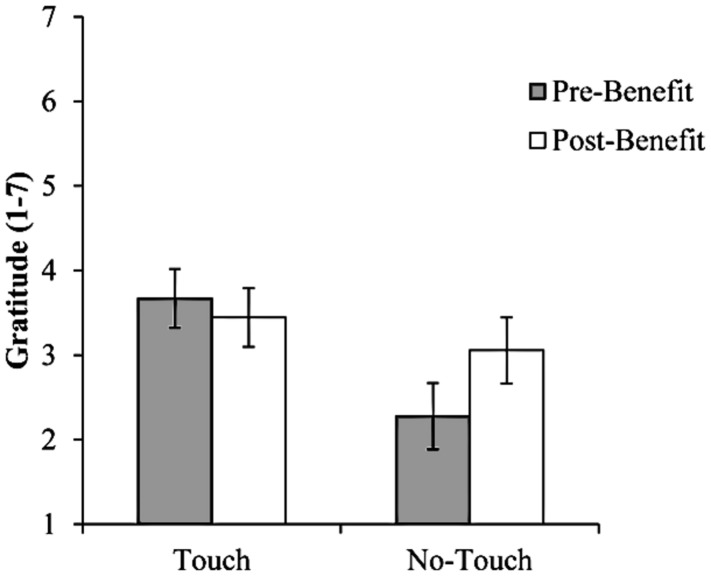
**Pre- and post-benefit gratitude as a function of touch condition, Study 1 (*N* = 36).** Error bars represent SEM.

The Touch × Benefit interaction was statistically significant, *F*(1,34) = 4.49, *p* = 0.042, η^2^ = 0.12. Simple comparisons per condition revealed that participants in the touch conditions did not show any differences between pre-benefit [*M* = 3.67, SE = 0.35, 95% CI = (2.96; 4.37)] and post-benefit gratitude [*M* = 3.44, SE = 0.39, 95% CI = (2.65; 4.24), *F* < 1, *p* > 0.250]. However, in the absence of touch, participants reported feeling more grateful after the benefit than before (pre-benefit gratitude: *M* = 2.28, SE = 0.35, 95% CI = (1.57; 2.98); post-benefit gratitude: *M* = 3.06, *SE* = 0.39, 95% CI = (2.26; 3.85), *F*(1,34) = 5.43, *p* = 0.026, see **Figure 1**).

##### The role of communal feelings: mediational analyses

**FIGURE 2:**
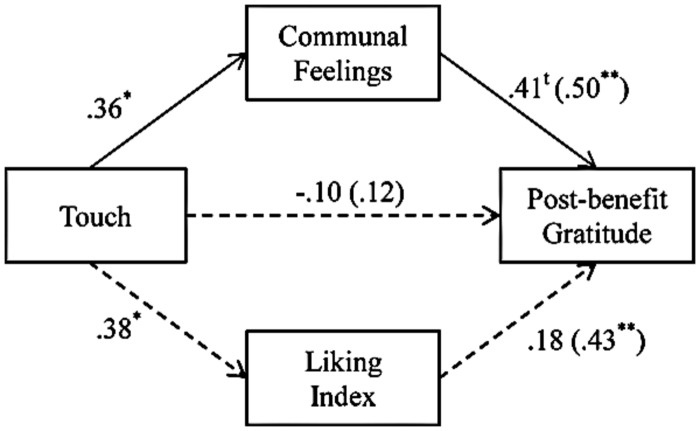
**Results of the regression analysis corroborating that the effect of touch on post-benefit gratitude is mediated by communal feelings (vs. liking) in Study 1.** The numbers are standardized regression coefficients. The numbers outside parentheses represent β-weights when all variables are used concurrently as predictors, whereas the numbers in parentheses are zero-order correlations. ^t^*p* < 0.10; ^∗^*p* < 0.05; ^∗∗^*p* < 0.01.

Given the significant effects of touch on liking and on communal feelings, we tested if either liking or communal feelings mediated the link between touch and post-benefit gratitude. We conducted a multiple mediation analysis using bootstrapping (Hayes and Preacher, 2014). We tested the mediation analyses with post-benefit gratitude as the dependent variable, entering touch as the independent variable and both communal index and liking as mediators. The results revealed that communal index, but not liking^3^, mediated the effect of touch on post-benefit gratitude. The indirect effect of touch on gratitude through communal index [effect value: 0.49, 95% CI (0.01; 1.46), *p* < 0.05] was statistically significant, whereas the indirect effect through liking for the confederate was not [effect value: 0.21, 95% CI (-0.21; 1.10), *p* = ns; see **Figure 2**].

Next, we tested the feedback models, by interchanging the mediators and the outcome variables (Baron and Kenny, 1986). In the feedback model, we conducted a multiple mediation model, using communal index as the dependent variable, and both post-benefit gratitude and liking as mediators. The only statistically significant indirect effect was through liking for the confederate [effect value of 0.48, 95% CI (0.13; 1.01), *p* < 0.05] and not through post-benefit gratitude [effect value of 0.01, 95% CI (-0.09; 0.41), *p* = ns]^4^. This result supports a causal interpretation of the predicted mediation effect.

**FIGURE 3:**
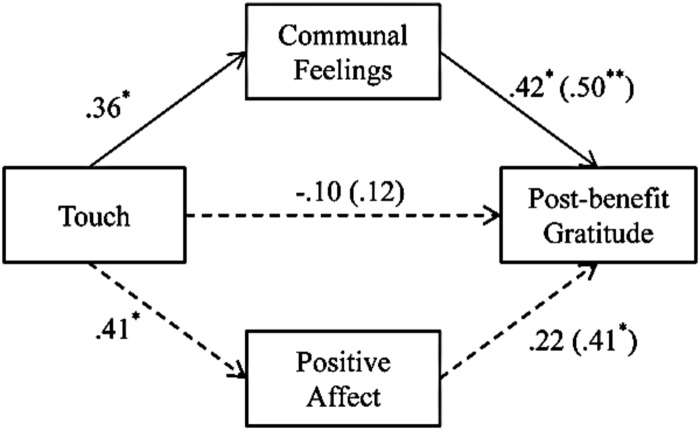
**Results of the regression analysis corroborating that the effect of touch on post-benefit gratitude is mediated by communal feelings (vs. positive affect) in Study 1.** The numbers are standardized regression coefficients. The numbers outside parentheses represent β-weights when all variables are used concurrently as predictors, whereas the numbers in parentheses are zero-order correlations. ^∗^*p* < 0.05; ^∗∗^*p* < 0.01.

In order to disentangle whether gratitude is an outcome of touch as indicator of communal relationships, and not a general prosocial outcome triggered by the positive affect of being touched in a friendly way, we repeated the multiple mediation analysis with positive affect instead of liking as potential alternative mediator of the touch ≥ gratitude link (following the procedure of Hayes and Preacher, 2014). We used touch as the independent variable, both positive affect and communal index as the mediators, and post-benefit gratitude as the dependent variable. As predicted, the indirect effect was statistically significant only through communal index, with an effect value of 0.55, 95% CI (0.11; 1.17), *p* < 0.05. The indirect effect through positive affect was not statistically significant [effect value: 0.09, 95% CI (-0.14; 0.47), *p* = ns; see **Figure 3**].

When we tested the feedback model entering communal feelings as the dependent variable and both positive affect and post-benefit gratitude as mediators, the significant indirect effect which emerged was through positive affect [effect value: 0.36, 95% CI (0.61; 0.98), *p* < 0.05], and not through post-benefit gratitude [effect value: 0.09, 95% CI (-0.12; 0.49), *p* = ns]. This is further evidence for the predicted causal chain via communal feelings over an alternative model via positive affect.

### Discussion

To summarize, our results show that a friendly touch increased gratitude toward the confederate. Moreover, in the absence of touch, benefits increased gratitude toward a confederate. A friendly touch also augmented communal feelings, and liking for a confederate, as well as general positive affect. However, only communal feelings, and neither liking, nor positive affect mediated the link between touch and post-benefit gratitude. The results of feedback models support a causal interpretation of this mediation. Even though these results confirm our initial hypotheses, we obtained them in a specific – communal – mindset. Thus, our second study serves two purposes: (1) to replicate the findings of the first study; and (2) to better understand the context where friendly touch increases gratitude and communal feelings.

## Study 2

### Materials and Methods

In Study 2, we induced a communal mindset in half the sample and a neutral one in the other half to test if the effect of a friendly touch depends on the mindset. Relational embodied cues can have different meanings in different contexts (e.g., Lakens et al., 2011), and touch is interpreted as more negative and unpleasant when a competitive (vs. cooperative) context is salient (Camps et al., 2012). Nevertheless, students at the same institution in Portugal generally meet each other in a friendly mindset. Thus, we expected the same effects of touch irrespective of the mindset condition.

#### Participants, Design, and Procedure

Ninety-two female participants contributed with data to this experiment, and 84 fulfilled our inclusion criteria, with a mean age of 21.00 (SD = 2.88). We calculated the sample size based on the estimated effect size (*d*) of 0.94 from Study 1, and to reach 80% of statistical power. Therefore our initially determined total sample size was 38. However, because we added one more factor with two cells, and in order to reach the 80% of statistical power for each mindset, we doubled our sample size. This way, we could be fairly confident to detect an unpredicted moderation by mindset if there was any. Therefore, we aimed to recruit approximately 90 participants. The design was a 2 (mindset: communal vs. neutral mindset) by 2 (touch: touch vs. no-touch) by 2 (benefit: pre-benefit gratitude vs. post-benefit gratitude) factorial design. Both mindset and touch were between-subjects conditions with random assignment to condition, and benefit was a within-subjects condition. The procedure followed exactly the one from Study 1, except that in the neutral (non-relational) mindset condition, the word “friendship” from the mindset induction was replaced by the words “the Universe.” Thus, participants were asked to write down five things or facts that define the Universe.

All procedures were conducted according to the ethical guidelines and approved by the ethics board of the Scientific Commission of the hosting institution, Centro de Investigação e Intervenção Social (CIS-IUL).

### Results

#### Touch and Pre-Benefit Gratitude

To replicate the findings from Study 1, we tested again the hypothesis that a friendly touch increases gratitude, regardless of the context, in a Touch × Mindset ANOVA on pre-benefit gratitude. As in Study 1, there was a significant main effect of touch, *F*(1,80) = 4.33, *p* = 0.041, η^2^ = 0.05. Regardless of the mindset, participants who received a touch reported feeling more pre-benefit gratitude [*M* = 3.73, SD = 1.76, 95% CI = (3.19; 4.27)] than participants who did not receive a touch [*M* = 2.93, SD = 1.71, 95% CI = (2.41; 3.47)]. Neither the main effect of the mindset, nor the interaction of Touch × Mindset were statistically significant, *F*s < 1, *p*s > 0.250.

#### Touch, Communal Feelings, Liking, and Affect

Furthermore, we analyzed the effect of touch and mindset on the communal feelings, liking, and positive affect indices, separately. There was a main effect of touch on the communal feelings index, *F*(1,80) = 4.91, *p* = 0.030, η^2^ = 0.06. In the touch condition participants felt more communal toward the confederate [*M* = 4.33, SD = 1.07, 95% CI = (4.00; 4.67)] than participants in the no-touch condition [*M* = 3.81, SD = 1.05, 95% CI = (3.49; 4.14)]. Neither the main effect of mindset, nor the interaction effect of Touch × Mindset were statistically significant, *F*s < 1, *p*s > 0.250.

As for the liking index, the results showed a significant main effect of touch on liking, *F*(1,80) = 5.85, *p* = 0.018, η^2^ = 0.07, but the main effect of mindset on liking was not statistically significant, *F*(1,80) = 3.13, *p* = 0.081, η^2^ = 0.04. The interaction of Touch × Mindset was not statistically significant, *F* < 1, *p* > 0.250. The effect of touch on liking indicates that participants who received a touch on the shoulder liked the confederate more [*M* = 4.83, SD = 1.19, 95% CI = (4.47; 5.20)] than participants who did not receive a touch on the shoulder [*M* = 4.21, SD = 1.17, 95% CI = (3.86; 4.57)]. Even though the effect of the mindset on liking for the confederate was not significant, the pattern was in the expected direction: participants in the communal mindset conditions liked the confederate more [*M* = 4.74, SD = 1.35, 95% CI = (4.39; 5.12)] than participants in the neutral mindset conditions [*M* = 4.29, SD = 1.04, 95% CI = (3.94; 4.65)].

Finally, we tested the effect of both touch and mindset on positive affect and on negative affect, individually. Unlike in Study 1, the main effect of touch on positive affect was not statistically significant, *F*(1,80) = 1.19, *p* = 0.280, nor was any of the other effects, *F*s < | 1.60|, *p*s > 0.210.

#### Touch, Communal Feelings, and Post-Benefit Gratitude

##### Benefits and gratitude

**FIGURE 4:**
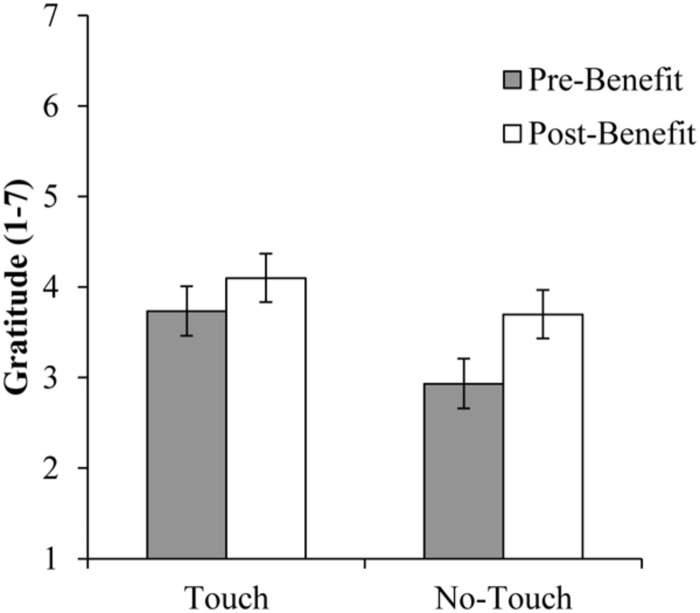
**Pre- and post-benefit gratitude as a function of touch condition, Study 2 (*N* = 84).** Error bars represent SEM.

In order to replicate the results from Study 1, we used a mixed models linear analysis of variance to test for the contrast analysis of the interaction effect of Touch × Benefit on gratitude. We tested the specific hypothesis that a friendly touch would increases gratitude, and that benefits would only increase gratitude in the no touch conditions. Thus, we coded the contrast as follows: touch – pre-benefit gratitude 0.25, touch – post-benefit gratitude 0.25, no touch – pre-benefit gratitude -0.75, no touch – post-benefit gratitude 0.25. The results showed a statistically significant interaction of Touch × Benefit, *t*(111.54) = 3.14, *p* = 0.002, contrast estimated difference = 0.68, 95% CI (0.25; 1.12), (see **Figure 4**). As predicted, the mean for no touch – pre-benefit gratitude was significantly smaller than the means in the other three conditions (see **Table 2** for descriptive statistics). Moreover, we tested if benefits increase gratitude beyond the level induced by touch alone. The contrast was coded as follows: touch – pre-benefit gratitude -0.5, touch – post-benefit gratitude 0.5, no touch – pre-benefit gratitude 0, no touch – post-benefit gratitude 0. No differences were found between pre- and post-benefit gratitude in the touch conditions, *t*(82) = 1.16, *p* = 0.250, suggesting that in the touch condition, the benefit did not increase gratitude.

**Table 2:** Means and SD as a function of touch, and correlations of pre- and post-benefit gratitude, positive and negative affect, communal feelings, and liking indices of Study 2 (*N* = 84).

Variables	Touch Mean (SD)	No touch Mean (SD)	1	2	3	4	5
1. Pre-benefit gratitude	3.73 (1.76)	2.93 (1.71)	–				
2. Post-benefit gratitude	4.10 (1.76)	3.70 (1.79)	0.35^∗∗∗^	–			
3. Positive affect	3.67 (1.39)	3.33 (1.48)	0.72^∗∗∗^	0.27^∗^	–		
4. Negative affect	1.44 (0.76)	1.27 (0.46)	0.14	0.13	0.08	–	
5. Communal feelings	4.33 (1.07)	3.81 (1.05)	0.54^∗∗∗^	0.42^∗∗∗^	0.51^∗∗∗^	-0.14	–
6. Liking index	4.83 (1.19)	4.20 (1.17)	0.46^∗∗∗^	0.19^t^	0.40^∗∗∗^	-0.10	0.67^∗∗∗^

^t^*p* < 0.10; ^∗^*p* < 0.05; ^∗∗∗^*p* < 0.001.

##### The role of communal feelings: mediational analyses

**FIGURE 5:**
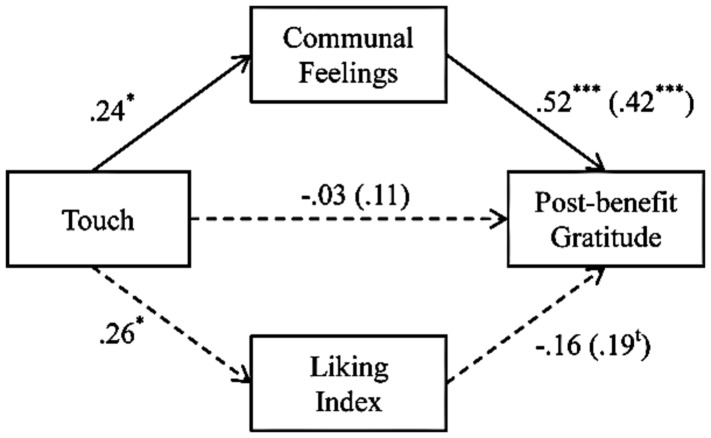
**Results of the regression analysis corroborating that the effect of touch on post-benefit gratitude is mediated by communal feelings (vs. liking) in Study 2.** The numbers are standardized regression coefficients. The numbers outside parentheses represent β-weights when all variables are used concurrently as predictors, whereas the numbers in parentheses are zero-order correlations. ^t^*p* < 0.10; ^∗^*p* < 0.05; ^∗∗∗^*p* < 0.001.

According to the results of Study 1, we tested again the same mediation analyses using post-benefit gratitude as the dependent variable. We entered touch as the independent variable and both communal feelings index and liking as mediators. The indirect effect through communal feelings index [effect value: 0.44, 95% CI (0.08; 1.03), *p* < 0.05] was statistically significant, however, the indirect effect through liking for the confederate was not [effect value: -0.15, 95% CI (-0.65; 0.08), *p* = ns; see **Figure 5**].

For the feedback model, we entered the communal index as the dependent variable, and both post-benefit gratitude and liking as mediators. As in Study 1, the indirect effect happened through liking [effect value of 0.33, 95% CI (0.06; 0.63), *p* < 0.05] and not through post-benefit gratitude [effect value of 0.07, 95% CI (-0.06; 0.24), *p* = ns]. This result supports a causal interpretation of the predicted mediation effect in Study 2.

**FIGURE 6:**
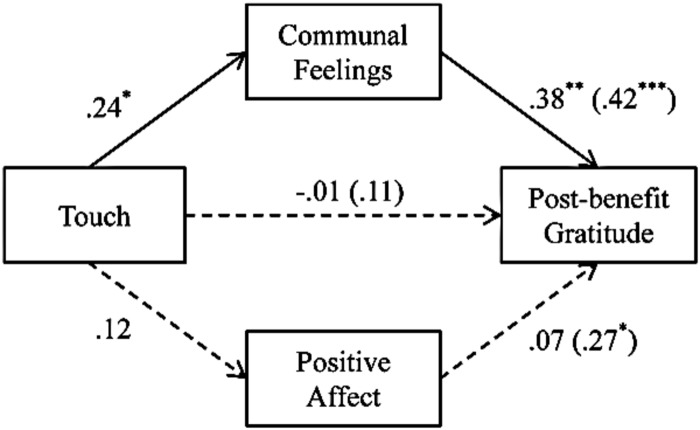
**Results of the regression analysis corroborating that the effect of touch on post-benefit gratitude is mediated by communal feelings (vs. positive affect) in Study 2.** The numbers are standardized regression coefficients. The numbers outside parentheses represent β-weights when all variables are used concurrently as predictors, whereas the numbers in parentheses are zero-order correlations. ^∗^*p* < 0.05; ^∗∗^*p* < 0.01; ^∗∗∗^*p* < 0.001.

Given our results from Study 1, we tested again gratitude as an outcome of communal feelings vs. positive affect, in order to replicate the predicted mediation pattern. We used touch as the independent variable, both positive affect and communal index as the mediators, and post-benefit gratitude as the dependent variable. The results showed that the indirect effect which was statistically significant happened through communal index on post-benefit gratitude [effect value of 0.32, 95% CI (0.06; 0.84), *p* < 0.05], and not through positive affect [effect value: 0.03, 95% CI (-0.07; 0.31), *p* = ns; see **Figure 6**].

When we tested the feedback model, the communal feelings variable was entered as the dependent variable and both positive affect and post-benefit gratitude were entered as mediators. Neither of the indirect effects were statistically significant [positive affect: effect value: 0.11, 95% CI (-0.07; 0.44), *p* = ns; post-benefit gratitude: effect value: 0.07, 95% CI (-0.05; 0.24), *p* = ns]. This result supports a causal interpretation of the predicted mediation effect.

### Discussion

Study 2 replicates the findings from Study 1: (1) a friendly touch increases gratitude and benefit increases gratitude in the absence of touch; and (2) a friendly touch increases post-benefit gratitude via communal feelings. Our mindset manipulation did not moderate these effects. Thus, we conclude that a friendly touch is interpreted as a communal signal in an explicitly friendship-oriented, but also in a more neutral mindset, and therefore produces gratitude.

## General Discussion

Two studies tested whether among university students, a brief friendly touch on the shoulder increases feelings of gratitude. Our results showed that those who had been touched by a cooperator indicated more gratitude toward her. Moreover, participants who had been touched felt more communal in relation to the cooperator and liked her better than those not touched. As predicted, only the communal index, and neither liking nor positive affect, mediated the link between touch and gratitude measured in the end of the study. Our results also showed that both touch and benefits augment gratitude, but benefits do not further increase gratitude beyond the level induced by touch alone. Given that touch signals communion (e.g., Fiske and Haslam, 2005), and gratitude is a response to communal intentions (Simão and Seibt, 2014), we suggest that a friendly touch is sufficient to induce gratitude among unknown interactions partners. These results highlight the positive contribution of touch for close social relationships. Furthermore, they showed that confederates in the touch condition were liked better and triggered more positive affect, but these effects were not responsible for the effect of touch on gratitude.

According to Algoe (2012), a responsive gesture evokes gratitude, which in turn helps identify a high-quality relational partner. We suggest that a friendly touch on the shoulder is probably perceived as such a responsive gesture because the receiver may perceive it as signaling attention, care, and consideration (see Reis et al., 2004). Another responsive gesture is intentionally responding to a partner’s needs by benefitting him or her (e.g., Algoe et al., 2008). Our findings also show that benefits are important to gratitude: In both studies we found that in the absence of touch, participants felt grateful toward the confederate after having received a benefit. In the presence of touch, the benefit did not boost gratitude beyond the level evoked by the touch alone.

Our mediation analyses showed that gratitude was increased by touch, to the extent that participants perceived the interaction with the partner as communal. Post-benefit gratitude was mediated by communal feelings, and not by liking for the interaction partner, nor by the positive affect induced by touch. Previous research found that communal feelings predict the amount of gratitude felt for a benefit, and they also predict gratitude in peer relationship over time (Simão and Seibt, 2014). Communal feelings are based on a communal sharing relational model that encompasses non-contingent benefits, kindness, union among relational partners and caring for one another. Individuals respond to each other’s needs and they are concerned with each other’s well-being (Fiske, 1992). Thus, we suggest that concrete benefits are not crucial for feeling grateful as previously suggested (e.g., McCullough et al., 2001), but they are probably perceived as communal cues (e.g., Fiske, 2004; Simão and Seibt, 2014), and should therefore, strengthen the perception of the relationship as communal in order to trigger gratitude.

## The Role of Gratitude in Communal Relations

Our findings show that the link between touch and gratitude was mediated by communal feelings, rather than by liking or by positive affect. This suggests that gratitude is important for the initiation and confirmation of communal relationships (see also Simão and Seibt, 2014). Relational models theory posits that touch, responsive benefits, and communal feelings are all aspects of the communal sharing model of relating to each other (Fiske, 1992). When individuals perceive the cues that embody the intention to relate in a communal way, such as touch, they activate the communal model (Fiske and Haslam, 2005; Schubert et al., 2008). Touch signals the intention to initiate or to maintain a communal relation, thus confirming and reinforcing the existence of a communal bond. Because individuals find communal relationships rewarding (Fiske, 1992), touch thus signals a person’s willingness to engage in this rewarding relationship. This confirmation thus can trigger communal feelings and gratitude toward the relational partner.

While the current findings and those of Simão and Seibt (2014) suggest that relational perception as communal predicts gratitude, another line of research also found that gratitude motivates individuals to invest in communal relationships (e.g., Algoe et al., 2008; Lambert et al., 2010). Feeling grateful toward a partner increases communal vs. individual gains in an economic exchange task (DeSteno et al., 2010), and it augments prosocial behavior toward strangers (Bartlett and DeSteno, 2006). Additionally, gratitude is suggested to strengthen communal relationships with an interaction partner by promoting social affiliation and social inclusiveness (Bartlett et al., 2012). Bringing these two lines of research together, we suggest that individuals need to activate the mental model of communal sharing in order to feel gratitude, and that gratitude in turn motivates to invest communally in the relationship (e.g., Algoe et al., 2008; Lambert et al., 2010; Algoe, 2012; Bartlett et al., 2012). It would be interesting if further research would test a full model of gratitude, taking into account the mental structure of communal sharing, and the communal motivation triggered by gratitude.

## Limitations and Implications for Further Research

Even though our results highlight the importance of communal feelings for feeling grateful, some limitations of the present studies need to be pointed out. Specifically, we acknowledge that we only measured communal feelings instead of manipulating them. To strengthen our interpretation of the present results as a causal chain, future studies should seek to manipulate communal feelings. Thus, a potential bi-directionality of communal feelings and gratitude cannot be excluded with the current data. Additionally, we also found that touch increased communal feelings and gratitude in neutral and communal mindsets. Accordingly, this effect seems to be independent of context. However, we did not include a manipulation check for the effectiveness of our mindset manipulation. Prior research contrasted the communal mindset induction (friendship) from an authority ranking mindset induction and found effects of authority ranking cues only in the latter mindset (Schubert and Giessner, in preparation). Given that touch can be a communal sharing (Fiske, 2004) or an authority ranking cue (Carney et al., 2005), depending on the context, this finding suggests that in our studies, touch acted as a communal signal. We chose the neutral, non-relational mindset induction (universe) for its face value and its similarity to the communal induction. It is possible, however, that focusing on the universe may have induced awe in some of our participants, which is more connected to authority ranking (Keltner and Haidt, 2003). Nevertheless, both mindsets are clearly non-negative.

In negative or competitive contexts, touch can be interpreted as more negative, and probably induce other emotions. Accordingly, receiving a touch in a competitive (vs. cooperative) context decreases helping behavior (Camps et al., 2012). We therefore assume that unfriendly touch decreases gratitude. Nevertheless, our main goal here was to understand the effects of touch in a non-negative setting. In such a setting, it seems to be perceived as a friendly gesture, a proxy for communal sharing and gratitude.

Furthermore, given that benefit and measurement time were confounded, it is possible that what we see as an effect of benefit is really an effect of measurement time. Future research should fully cross these variables. An additional limitation is that we only had female participants. There are many gender dynamics in physical touch processes, but to model them accurately and precisely would require a much larger sample. Thus, further research is needed to confirm the same effect of touch on men. While our reasoning should apply to both genders, factors that co-vary with gender such as the cultural appropriateness of touch or sexual connotations of touch likely moderate its effects by influencing its interpretation as a responsive and considerate gesture.

Another limitation concerns the ecological validity of our findings. We found that touch and benefits increase gratitude in minimal relationships with subtle manipulations. In natural interactions, (1) individuals typically know each other and have a relationship from before, (2) touch is often more intensive than the type used in this study, as in hugging, kissing, or holding hands, and (3) benefits given in a communal relationship also can be quite large, such as sharing income, or dedicating a lot of time to help the other person. All of this suggests that the effects of gratitude and benefits may be even larger in natural interactions than in our laboratory studies. However, natural interactions also differ from each other on many dimensions, suggesting that the influence of any one factor may be much harder to detect. In order to apply the present findings, it is therefore important to replicate them in field studies.

## Conclusion

Gratitude can improve well-being by increasing the appreciation of positive things in life (Wood et al., 2010) and increasing resilience to negative things (Fredrickson, 2013). Furthermore, communal relations and the support they offer are central for well-being and physical health (e.g., Emmons and McCullough, 2003). In the present research, we discovered that a friendly touch can promote gratitude and communal relations concurrently. We hope this will inform theorizing and practical implications for improving people’s health and well-being.

## Author Contributions

All the authors developed the study concept, and contributed to the study design. Testing and data collection were performed by CS. CS and BS performed the data analysis and interpretation. CS drafted the manuscript and BS provided critical revisions; all authors approved the final version of the manuscript for submission.

## Conflict of Interest Statement

The authors declare that the research was conducted in the absence of any commercial or financial relationships that could be construed as a potential conflict of interest.

## Footnotes

1 Initially we estimated the expected effect size based on the subsample of female patrons evaluating female servers from Hornik’s (1992) paper, (*N* per cell = 15), which seemed closest to our manipulation, measures, and sample composition (female–female dyads). However, because that subsample was small (and thus the effect size estimate has a large confidence interval), we decided to calculate the expected effect size based on this study plus two more studies, which we picked because they had similar independent and dependent variables to our study, yet a different sample composition: Erceau and Guéguen (2007) and Guéguen et al. (2010). The dependent variables from these studies that we used for deriving a combined estimate were friendliness (Erceau and Guéguen, 2007, *N* per cell = 20), compliance (Guéguen et al., 2010, average *N* per cell = 158), and the evaluation of a female server by female and male patrons (Hornik, 1992, *N* per cell = 30). We found an overall effect size (*d*) of 1.20. This is similar and even slightly larger than the effect size we based our sample size calculation on (*d* = 0.99). These estimates may seem large, however, because touch is an important interpersonal signal, individuals may be particularly sensitive to it, leading to large effects. Even though this suggests that the power of Study 1 was adequate, we decided to replicate the effect of Study 1, by conducting Study 2 with a larger sample size for the touch manipulation to increase our power and to decrease the likelihood of making a Type I error when interpreting the results.

2 Given that we did not have specific predictions for the post-benefit positive and negative affect items, we did not include them in the final analyses. Throughout this article, we use *Positive Affect* and *Negative Affect* to refer to the pre-benefit measurement.

3 Given that communal and liking indices were highly correlated in Study 1 (*r* = 0.70) and in Study 2 (*r* = 0.67), we tested for multicollinearity of these two indices as predictors of post-benefit gratitude. We applied the rule of thumb for Variance Inflation Factor (VIF) and tolerance, based on the values suggested by O’Brien (2007). A VIF of 10 or higher, and a tolerance value of 0.25 or lower indicate excessive or serious multicollinearity between the two predictors. For Study 1, when regressing post-benefit gratitude on the communal and liking indices, the collinearity statistics indicate a VIF of 1.95, and a tolerance of 0.51. The same analysis was repeated for Study 2, with a VIF of 1.81, and a tolerance of 0.55. Thus, we are confident to interpret the path models of **Figures 2** and **5**.

4 Additional mediational analyses interchanging liking for the confederate and post-benefit gratitude for Studies 1–2 can be consulted in the Supplementary Material.
