# Supplementary material for: Friendly touch increases gratitude by inducing communal feelings
Source: Front Psychol. 2015 Jun 15;6:815. doi: 10.3389/fpsyg.2015.00815 (PMC4467067; doi:10.3389/fpsyg.2015.00815)
Supplement: Supplementary file 1 [file Table_1.DOCX]

Table S1. Criteria for exclusion, assessment and number of participants excluded according to each criterion for both Study 1 and Study 2.

| Criterion for exclusion | Assessment | N excluded in Study 1 | N excluded in Study 2 |
| --- | --- | --- | --- |
| Outliers | Using the procedure suggested by Field (2009, p. 97), we graphed the data of all the main dependent variables with a boxplot: communal sharing, synchrony, and rapport scale, liking index, and pre- and post-benefit gratitude. | 0 | 3  Two in the synchrony scale and one in the communal sharing scale. To deal with these outliers, we followed what was suggested by Field (2009, p. 153), and we removed these three cases from the main analyses. |
| Suspicion that the other participant was a confederate | Oral probe for suspicion:  - Did you notice anything strange during the experiment  - Did you notice anything strange about the other participant? | 0 | 4 |
| Suspicion of the hypothesis | Written and oral probe for suspicion:  - What do you think the study was about? | 0 | 1 |
