# Supplementary material for: Friendly touch increases gratitude by inducing communal feelings
Source: Front Psychol. 2015 Jun 15;6:815. doi: 10.3389/fpsyg.2015.00815 (PMC4467067; doi:10.3389/fpsyg.2015.00815)
Supplement: Supplementary file 2 [file Data_Sheet_1.DOCX]

Additional mediation analyses for Study 1

In order to interchange all the mediators in the multiple mediation model, we tested an extra feedback model, using liking for the confederate as the dependent variable, and both communal index and post-benefit gratitude as the mediators. The results were similar to the previous model: there was a significant indirect effect through communal index on liking for the confederate (effect value of 0.50, 95% CI [.11; 1.16], *p* < .05) and not through post-benefit gratitude (effect value of 0.03, 95% CI [-.05; .33], *p* = ns).
