# Supplementary material for: Friendly touch increases gratitude by inducing communal feelings
Source: Front Psychol. 2015 Jun 15;6:815. doi: 10.3389/fpsyg.2015.00815 (PMC4467067; doi:10.3389/fpsyg.2015.00815)
Supplement: Supplementary file 3 [file Data_Sheet_2.DOCX]

Additional mediation analyses for Study 2

We tested another feedback model, interchanging the liking for confederate and post-benefit gratitude. Thus, we used liking for the confederate as the dependent variable, and both communal index and post-benefit gratitude as the mediators. The results showed that the indirect effect was through communal index (effect value of 0.40, 95% CI [.06; .77], *p* < .05) and not through post-benefit gratitude (effect value of -0.03, 95% CI [-.20; .02], *p* = ns).
